# Nonlinear effects of meteorological factors on foodborne disease outbreaks in yantai from 2014 to 2024

**DOI:** 10.1038/s41598-025-22928-w

**Published:** 2025-11-10

**Authors:** Youxia Chen, Zhonghao Yuan, Guiqiang Wang, Jianping Wang, Fengguang Dong, Yiyi Zhang, Guiqin Sun, Xueying Feng

**Affiliations:** 1 Department of Nutrition and Food Hygiene, Yantai Center for Disease Control and Prevention, Yantai, 264003 China; 2 Shandong Key Laboratory of Marine Ecological Restoration, Shandong Marine Resource and Environment Research Institute, Yantai, 264006 China

**Keywords:** Foodborne diseases, Temperature, Precipitation, Wind speed, DLNM, Public health, Environmental impact, Epidemiology, Risk factors

## Abstract

Meteorological factors are of crucial importance in foodborne disease (FBD) outbreaks, and more quantitative research is required to elucidate the underlying mechanisms. This study aimed to evaluate the impact of meteorological factors on FBD outbreaks in Yantai from 2014 to 2024, using data reported by the Yantai Foodborne Disease Surveillance Network. The distributed lag non-linear model (DLNM) was employed to model the exposure-response relationships between temperature, precipitation, wind speed, and FBD outbreaks. Prior to model fitting, multicollinearity among variables was addressed via the variance inflation factor (VIF). Results demonstrated non-linear relationships and complex correlations between these variables. Higher temperatures, greater precipitation, and slower wind speeds were associated with an increased risk of FBD outbreaks. In the lagged time dimension, temperature had an immediate effect that decayed after 2–3 weeks; the maximum lagged risk for precipitation occurred within 1–3 weeks; and wind speed showed subdued fluctuations. The lag patterns of high-value and low-value effects of different elements exhibited symmetry. These findings underline the necessity of integrating meteorological monitoring into FBD surveillance systems in accordance with their respective impact patterns.

## Introduction

Foodborne diseases (FBD), encompassing any illnesses arising from the consumption of spoiled or contaminated food with disease-causing bacteria, viruses, parasites, toxins, pesticides, or drugs, pose a significant challenge to public health, and can also impede socioeconomic development by adversely affecting national economies, tourism, and trade^1,2^. The World Health Organization (WHO) has reported that approximately 600 million people globally suffered from FBD each year^2^. Despite the fact that various countries were vigorously promoting the establishment of FBD surveillance networks, it was estimated that only a small fraction of these cases were currently recorded^3^. Additionally, the economic burden of FBD cannot be overlooked, because the World Bank estimated that the annual productivity loss due to unsafe food in low- and middle-income countries amounted to around $95 billion per year^4^.

FBD-inducing factors existed throughout the food chain, from primary production to final consumption, and their transmission routes were complex, involving multiple FBD pathogens as well as chemical and biological toxins^3,5^. Notably, numerous pathogens exhibited strong environmental persistence, which was especially crucial in the context of climate change. Reports have indicated significant associations between meteorological factors and FBD outbreaks^6^.

In recent decades, climate change and the rise in extreme weather events may further exacerbate this vulnerability, thus affecting the incidence of diseases such as food- and water-borne diseases^5,7–9^. Temperature, as an important indicator of climate sensitivity, has been incorporated into numerous studies on disease burden analysis^10,11^. A study in Europe indicated that warmer temperature and longer summer might lead to an increase in FBD outbreaks^6^. Moreover, the relationship between temperature and FBD, such as Salmonella and Campylobacter, was non-linear^8,12–14^. Besides temperature, altered precipitation conditions have also been associated with the onset of various FBD^15,16^, especially in some extreme precipitation events^17,18^. Some studies have proposed that extreme precipitation may be more likely than extreme heat to trigger infections such as Salmonella pathogens^19^. Alarming is the fact that some pathogens have demonstrated strong environmental adaptations and high stress tolerance to temperature change^20–22^. Compared with temperature and precipitation, the impact of wind speed was more complex and contradictory. Existing studies have shown that high wind speed can reduce the incidence rate^23^, while some other studies have found that high wind speed may increase the risk^24^. Such contradictions may result from differences in study areas and climatic conditions. A series of studies kept confirming that changes in climatic conditions may impact the incidence and transmission patterns of FBD outbreaks.

Whilst some researches on single factors or a specific kind of FBD have been carried out^25,26^, far too little attention has been paid to comprehensive meteorological impact on FBD outbreaks. It has been showed that the spatial and temporal patterns of the effects of climatic conditions on FBD outbreaks varied across different regions^15^. Therefore, this study aimed to explore the potential mechanisms between meteorological elements and FBD outbreaks, using a multi-year surveillance data reported by the FBD Surveillance Network combined with local meteorological conditions. In this paper, temperature, precipitation and wind speed were selected as representative elements to analyze the response of FBD outbreaks under different levels of exposure, thereby providing a scientific basis for further improving the design and implementation of public health sector interventions to address FBD.

## Materials and methods

### Study area and data collection

**Fig. 1:**
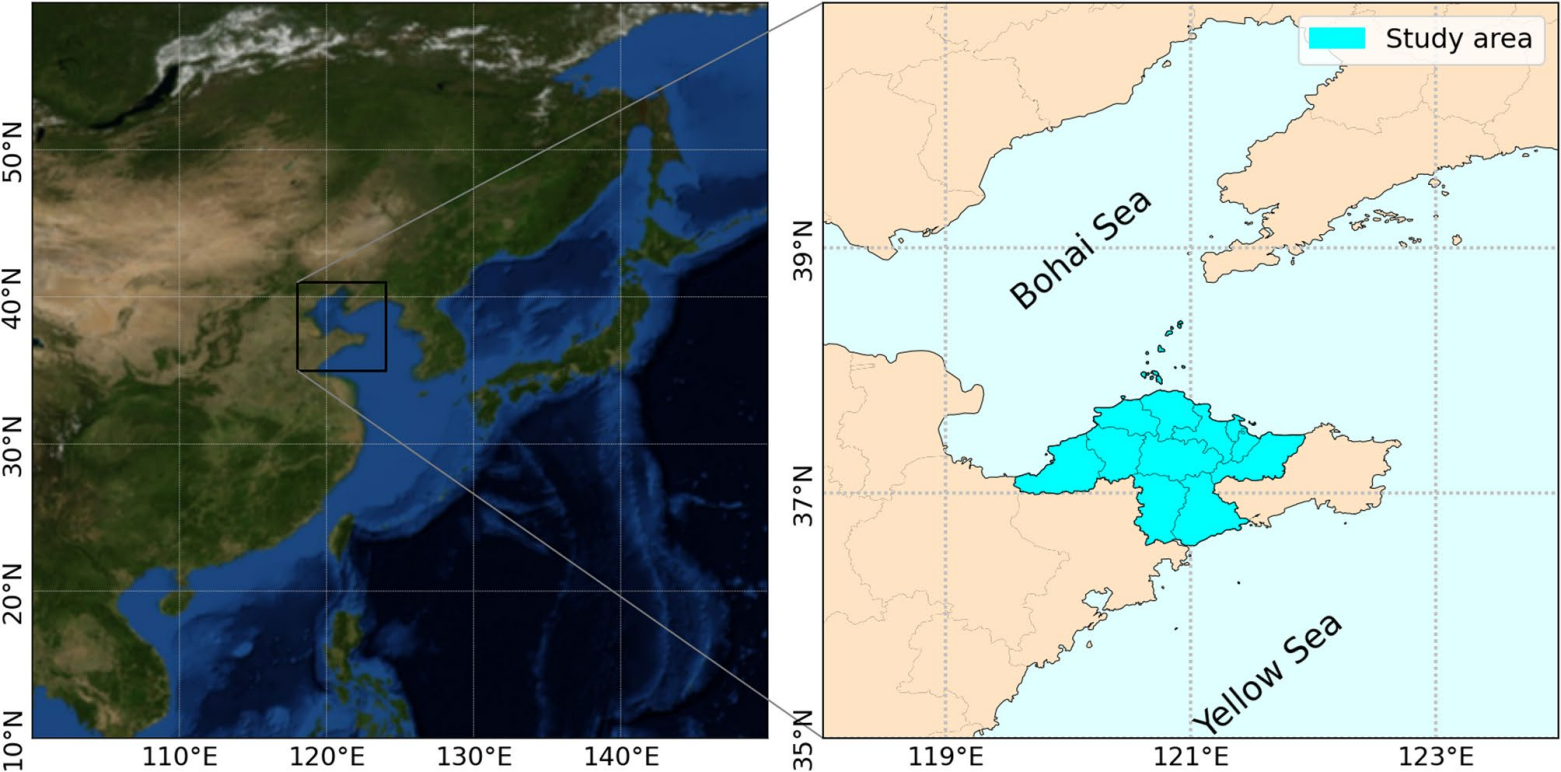
Study area. The left map was generated by Basemap (Version: 1.4.1, https://matplotlib.org/basemap/stable/users/introduction.html), and the right vector data was sourced from the National Geomatics Center of China through Tianditu (approval number GS(2024)0650, https://cloudcenter.tianditu.gov.cn/administrativeDivision).

We choses Yantai city as the study region (Fig. 1), a coastal city in the northeastern part of the Shandong Peninsula, East China, with a permanent population of approximately 7.03 million. Yantai has established a surveillance network for FBD that encompassed all 14 counties and municipalities, covering 167 sentinel hospitals, over 1000 community health service stations, and village health offices. Based on this system, it was possible to effectively obtain the status of relevant diseases in the region. In the system, the FBD outbreaks are defined as the occurrence of two or more similar diseases caused by the ingestion of a certain food. And the data used in this study were FBD outbreaks from July 2014 to March 2024.

The meteorological data were collected from the fifth-generation European Centre for Medium-Range Weather Forecasts (ECMWF) reanalysis data for the global climate and weather (ERA5) at https://cds.climate.copernicus.eu/datasets. ERA5 can be applied in multiple fields, including climate research, weather analysis, climate model validation, and environmental monitoring^27^. In this paper, the 2 m-temperature, total precipitation, 10 m-u- and 10 m-v-component of wind speed, and other meteorological data were utilized. The dataset had a temporal resolution of 1 h and a spatial resolution of 0.25 degree. Subsequently, the dataset was processed into weekly data in accordance with the requirements of the assessment calculation. The weekly average method was employed for temperature and wind speed, while the cumulative method was applied for precipitation.

## Methods

Related studies have demonstrated that environmental factors like temperature, rainfall, and vegetation cover usually typically have a non-linear and lagged effect on FBD outbreaks^26,28^. Therefore, in this paper, the distributed lag non-linear model (DLNM) was employed, which is a flexible modeling framework for exploring lagged effects. It has wide applications in many fields, including epidemiology, meteorology, and economic forecasting^16,29,30^. In the study, we took the Generalized Linear Models (GLM) as a framework and used DLNM to construct cross-basis functions for each meteorological factor. Then the exposure-response associations between FBD outbreaks and each element was explored. And the basic computational model adopted in this study can be written as:

1 $$CB(x,lag)={B_1}(x) \otimes {B_2}(lag)$$

2 $${y_t}=\alpha +\sum\limits_{{l=0}}^{L} {CB({x_{t - l}},l)} +g(t)+\sum\limits_{{p=1}}^{P} {{v_p}({z_p})}$$

In the Eq. (1), *CB*(*x*,* lag*) denoted the cross-basis function, which captured the effects of a specific exposure variable *x* across different lag times lag. And ⊗ represented the Kronecker product. For the horizontal feature dimension (*B*_*1​*_), a natural cubic spline (NS) model was utilized, while a fourth-order polynomial model was employed for the vertical lag dimension (*B*_*2*_​). In the Eq. (2), *t* represented the time index; *y* represented the count of FBD outbreaks weekly; *α* was the intercept (constant term), and *x* represented certain meteorological element, *l* was the lag, and *L* denoted the maximum lag. The function *g*() was used to eliminate seasonal and long-term trends and can be defined by a spline curve; *z* represented potential confounders, *P* was the total number of confounders, and the function *v*() was used to control the mutation effect of *z*. In this context, *v*() is specified as the NS, and its degree of freedom was set to 4. According to related research results^26,31,32^, the maximum lag length was set to 4 weeks (28 days). Meanwhile, we took the median value of each meteorological data as the baseline reference.

Disparate meteorological elements may be correlated with one another. In regression analysis, if elements with high linear or near-linear correlation were included in the confounding term, the multi-collinearity problem may be triggered. This problem might lead to more unstable estimation results and reduce the explanatory power of the independent variable on the dependent variable. Therefore, we adopt the variance inflation factor (VIF) to detect and measure multi-collinearity:

3 $$VI{F_{\text{j}}}={\raise0.7ex\hbox{$1$} \!\mathord{\left/ {\vphantom {1 {1 - \mathop R\nolimits_{j}^{2} }}}\right.\kern-0pt}\!\lower0.7ex\hbox{${1 - \mathop R\nolimits_{j}^{2} }$}}$$

In the Eq. (3), *R*^2^ represented the coefficient of determination of variable *j*, which measured the degree of linear correlation with others. A larger value of the *VIF* indicated a stronger linear correlation. Based on research experience, we set a threshold value of 10, and stepwise regression was applied to reduce multi-collinearity in the model for establishing the optimal subset of features. That was, in a dataset composed of multiple variables, if there are variables with VIF exceeding the threshold, the variable with the largest VIF was removed each time until the condition was met. After iterative optimization, precipitation was retained as confounders in the temperature-lag model; temperature was retained as confounders in the precipitation-lag model; and precipitation was retained as confounders in the wind speed-lag model. The above VIF calculations were completed based on the “statsmodels” library in Python software (version 3.9).

In the subsequent part of this paper, we developed DLNMs for different meteorological factors and analyzed the response mechanism between each meteorological factor and FBD outbreaks individually. We used the median value as the reference value, that is, its relative risk (RR) is 1.0. The analysis was completed with the “dlnm” package in R software (version 3.6.3).

### Ethics approval

The study was approved by Yantai Center for Disease Control and Prevention. Since the aggregated data used in the study were from routine surveillance and did not involve personal information, informed consent for study participation was not required.

## Results

### Description of FBD outbreaks and meteorological factors

From July 2014 to March 2024, Yantai reported 1,263 FBD outbreaks, with a cumulative 5,715 illnesses, having a weekly average of 11.31. Among the identified causative agents, 41.73% were biological bacteria such as Vibrio parahaemolyticus and Salmonella, 27.44% were fungi and their toxins like muscarinic toxins, 22.93% were toxic plants and their toxins such as saponins and hemagglutinins, 4.89% were chemical factors like nitrites, 2.63% were toxic animals and their toxins such as histamine and river herring toxins, and 0.38% were biological viruses such as norovirus.

**Fig. 2:**
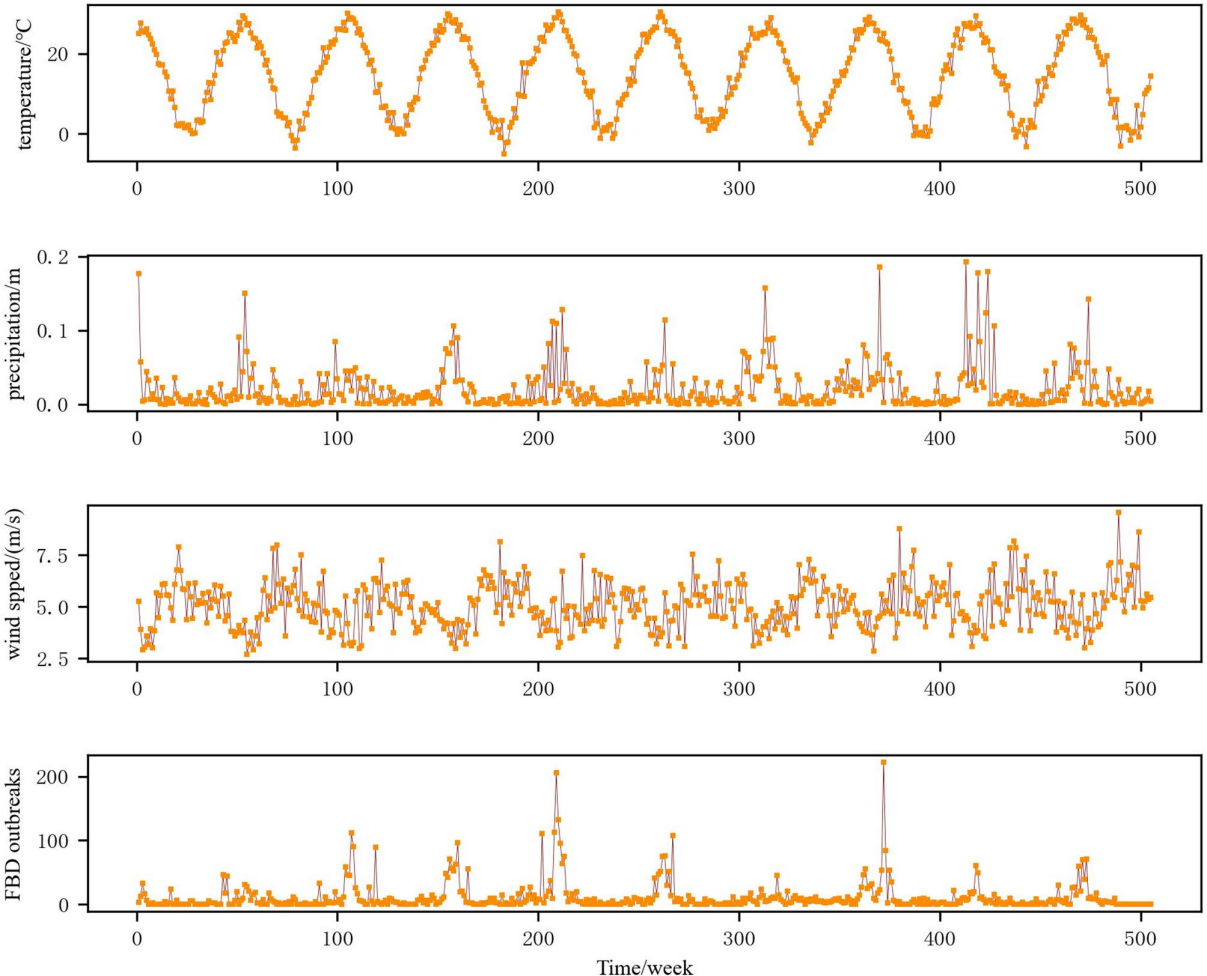
Time-series of meteorological factors and weekly reported FBD outbreaks.

**Fig. 3:**
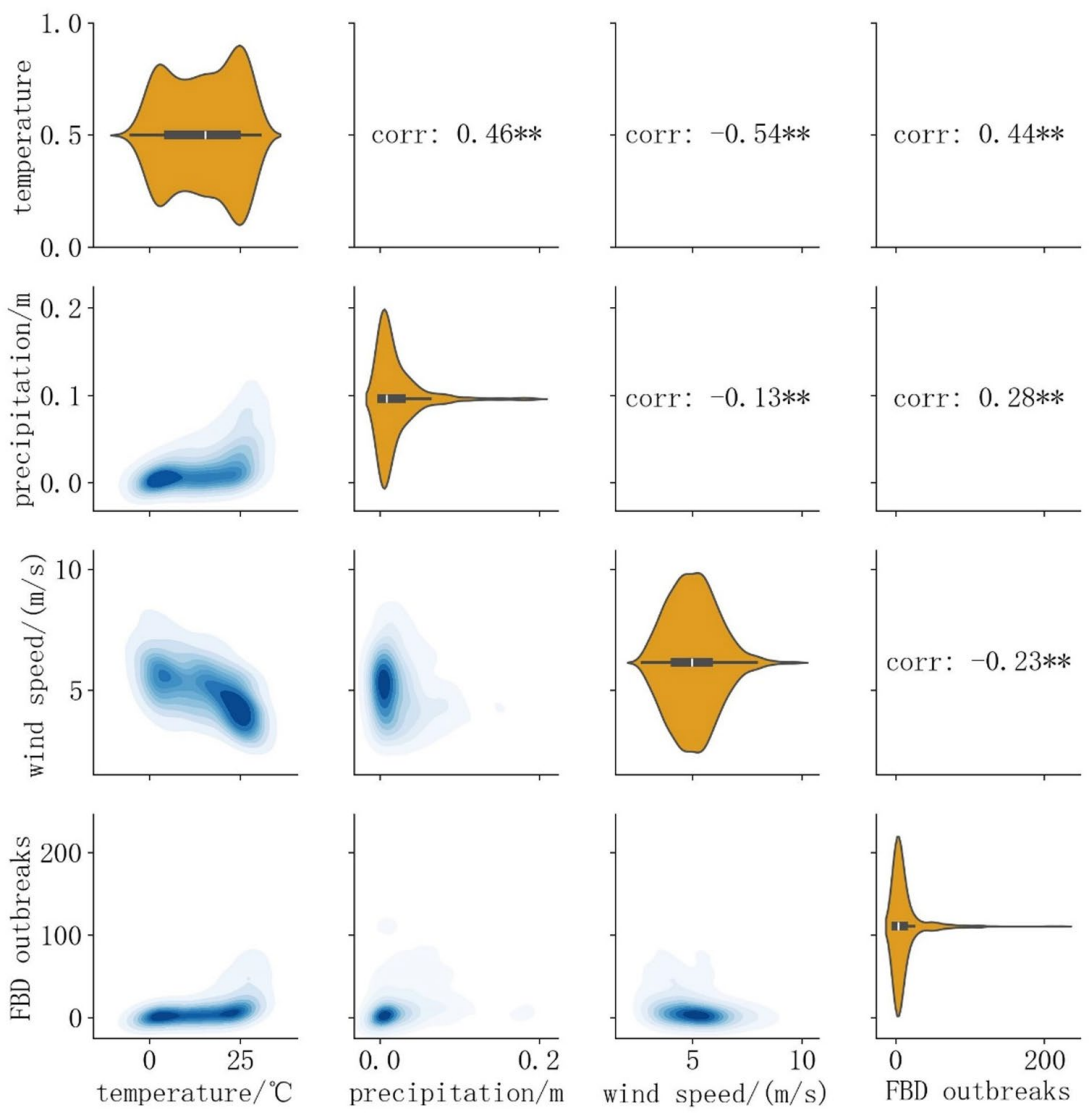
Kernel density distribution (KDE) and correlation coefficients for various meteorological data. The univariate violin plots are on the diagonal, the KDE contours and Pearson correlation coefficients between two-by-two variables are on the lower left, and the upper right individually, where ** denotes highly significant (*p* < 0.01) and * denotes significant (*p* < 0.05). Note that logarithmic operations have been applied to FBD outbreaks in the figure to compress the data range for visualization.

The time-series of diverse meteorological data and the distribution of the reported count of FBD outbreaks in Yantai was presented in Fig. 2. Evidently, temperature, precipitation, and wind speed had distinct seasonal cycles. Based on the data distribution (Fig. 3), temperature exhibited double-peak characteristics, while wind speed and precipitation showed single-peak and prominent trailing characteristics. Figure 3 indicated a certain correlation among the various types of data, and the correlation coefficients in the figure have passed the significance test at α = 0.01. This also indicated the necessity of conducting VIF screening before modeling.

### Temperature - fbd outbreaks response

**Fig. 4:**
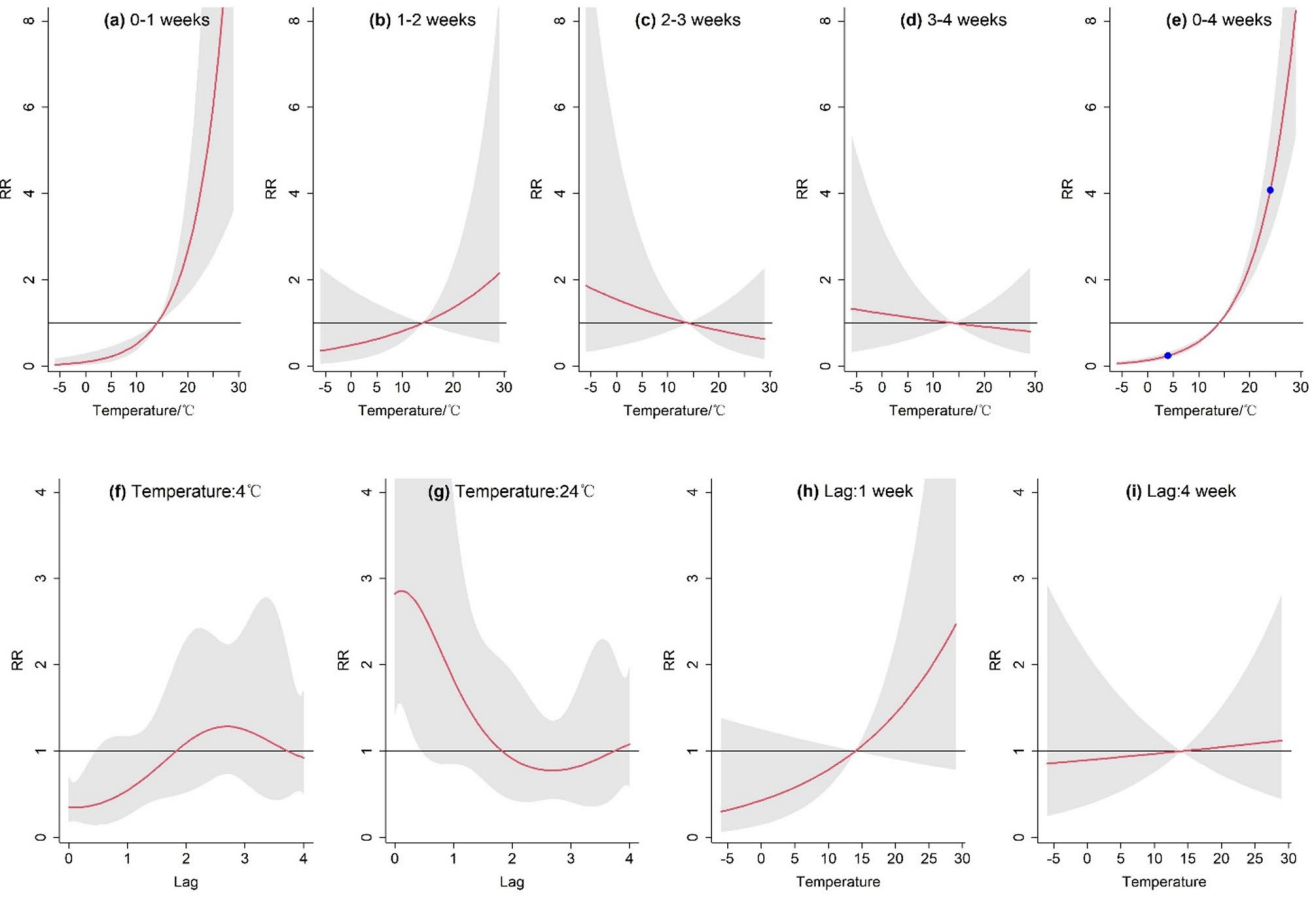
Exposure response between temperature (°C) and FBD outbreaks. The red lines are the mean RR, and the shaded areas indicate 95% confidence intervals. (a-e) Cumulative RR for Lag 0–1, 1–2, 2–3, 3–4, 0–4 weeks. In subfigure e, the scatter points are used to indicate the selected observed values. (f-i) Effect of specific temperature or time lags on the RR.

What is notable in Fig. 4 is that the cumulative disease risk increased as the mean weekly temperature rose. For an average weekly temperature of 24 °C and 4 °C, the 4-week cumulative lagged RR (Fig. 4e) was 0.25 (95% CI: 0.18–0.33) and 4.08 (95%CI: 3.05–5.44), respectively. However, this increasing trend was non-linear, with different evolutionary patterns in different lag time periods. At lag 0–1 weeks, the outbreak risk increased exponentially with rising exposure temperature. During lag 1–2 weeks, the increasing trend decreased significantly, and it reversed during lag 2–4 weeks. Judging from the overall cumulative RR within 0–4 weeks, when temperature was lower than the reference value (14.35 °C), the RR was less than 1.0, indicating an inhibitory-protective effect; when it was higher than the reference value, the RR continued to rise, meaning that the risk of FBD outbreaks increased rapidly and the temperature had a stimulatory-promotional effect.

Since excessively high or low temperature exposures led to opposite lagged effects, we selected ​​4 °C and 28 °C as representative values for low- and high-temperature exposures​​, respectively. The low-temperature effect had an immediate impact on FBD outbreaks in the population; strong low temperature significantly reduced the pathogenicity risk at lag 0 week (RR: 0.35, 95% CI: 0.18–0.70). However, this effect gradually returned to normal as time passed, forming a local maximum around lag 2.6 weeks (RR: 1.29, 95% CI: 0.74–2.25). High- temperature exposure showed the opposite trend, with the RR reaching a maximum at lag 0 (RR: 2.82, 95% CI: 1.42–5.59), and the heat-induced risk declined rapidly as the lag time increased, reaching a local minimum again around lag 2.6 weeks (RR: 0.78, 95% CI: 0.45–1.36).

### Precipitation - FBD outbreaks response

**Fig. 5:**
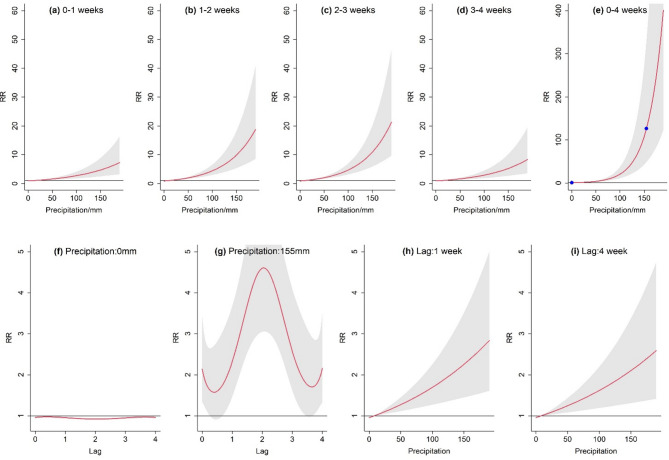
Exposure response between precipitation (mm) and FBD outbreaks. The red lines are the mean RR, and the shaded areas indicate 95% confidence intervals. (a-e) Cumulative RR for Lag 0–1, 1–2, 2–3, 3–4, 0–4 weeks. In subfigure e, the scatter points are used to indicate the selected observed values. (f-i) Effect of specific precipitation or time lags on the RR.

A nonlinear relationship between precipitation and FBD outbreaks was also observed in Fig. 5. In contrast to the pathogenic effects of temperature, precipitation exhibited a similar cumulative RR evolutionary pattern over time. Greater precipitation amounts lead to a higher disease risk. The primary difference lies in the rate of increase, which was slower during lag 0–1 weeks and lag 3–4 weeks, and significantly accelerated during lag 1–3 weeks.

Compared with the 4 weeks cumulative RR (Fig. 5e) of median weekly precipitation (8 mm, RR = 1.0), extreme precipitation (155 mm) significantly increased the risk (RR: 126.65, 95% CI: 48.68–329.47.68.47). For the 4 weeks cumulative RR, fewer precipitation events (0 mm) led to a 23% reduction in RR (RR: 0.77, 95% CI: 0.73–0.81). Taking the cumulative impact of 155 mm precipitation as an example, the cumulative RR at lags of 0–1, 1–2, 2–3 and 3–4 weeks were 4.97 (95% CI: 2.59–9.54), 10.67 (95% CI: 5.68–20.16), 11.82 (95% CI: 6.28–22.24) and 5.54 (95% CI: 2.79–11.01), respectively, with the lagged maximum of RR occurring during lag 1–3 weeks. Although extreme and sparse precipitation pose different outbreak risks in lag periods, they showed symmetrical characteristics in terms of the overall trend. Specifically, the overall trend of cumulative RR change showed a “low-high-low” or “high-low-high” pattern, peaking near lag 2 weeks at 0.92 (95% CI: 0.90–0.94) and 4.60 (95% CI: 3.05–6.93), respectively.

### Wind speed - FBD outbreaks response

**Fig. 6:**
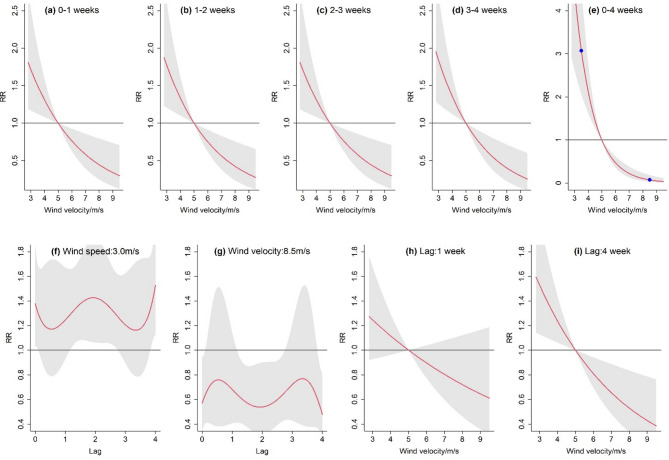
Exposure response between wind speed (m/s) and FBD outbreaks. The red lines are the mean RR, and the shaded areas indicate 95% confidence intervals. (a-e) Cumulative RR for Lag 0–1, 1–2, 2–3, 3–4, 0–4 weeks. In subfigure e, the scatter points are used to indicate the selected observed values. (f-i) Effect of specific wind speed or time lags on the RR.

The cumulative outbreak risk increased as the weekly mean wind speed increased (Fig. 6). For wind speeds of 3.5 m/s and 8.5 m/s, the 4-week cumulative lagged RR (Fig. 6e) were 3.09 (95% CI: 2.07–4.63) and 0.07 (95% CI: 0.03–0.18). Lower speed led to a higher disease risk, but the inhibitory effect of high wind speed diminished with cumulative time. When exploring the time-lagged effects of wind speed on FBD outbreaks, a remarkable pattern was observed: the cumulative pathogenic risk evolutionary showed high concordance across different lags, in contrast to the cumulative risk changes in temperature and precipitation. This phenomenon indicated a more homogeneous pathogenicity-risk distribution in the time dimension; that is, the wind speed effect was more stable and homogeneous in the short and long term.

Under high or low wind speed conditions, the disease risk evolution process was symmetrical. For example, under low wind speed (3.5 m/s), the RR exhibited a W-shaped evolution with an overall risk increase (RR > 1.0), and the maximum values were obtained near lag 0, lag 2, and lag 4 week, respectively. Under high wind speed (8.5 m/s), the evolution trend was M-shaped, and the overall risk was suppressed (RR < 1.0), with the minimum values also occurring near lag 0, lag 2, and lag 4 week. Despite the more convoluted evolution, the RR fluctuation ranges were relatively low under both wind speed conditions, further indicating that the wind speed induced risk was more stable in the time-lagged dimension.

### Distribution of meteorological factors for different outbreak levels

**Fig. 7:**
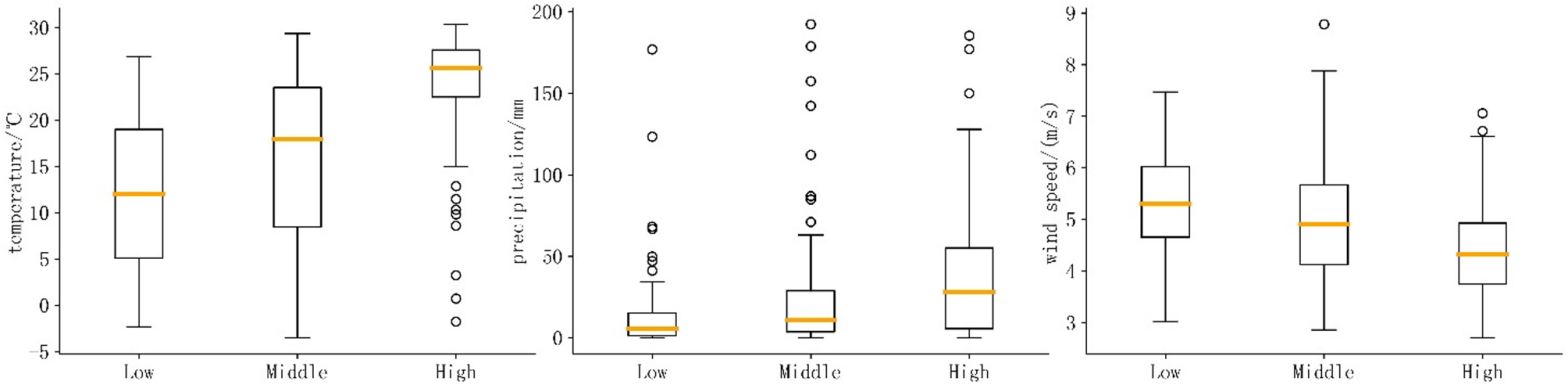
Box plots of the distribution of meteorological factors under different levels of FBD outbreaks. From left to right in order: temperature, precipitation, and wind speed. Black circles denote outliers, and bold orange lines indicate the median.

FBD outbreaks can be grouped into high, medium, and low categories based on the percentile of the number of outbreaks (the percentage split was set at 33%, 66%), for the purpose of studying the distribution of factors at different incidence levels (Fig. 7). A significant stratification phenomenon was captured; that was, outbreaks exhibited hierarchical discrepancy among groups. Principally, higher temperature, more abundant precipitation, and lower wind speed generally corresponded to higher frequencies of FBD outbreaks. The above results highlighted the crucial role of meteorological conditions in FBD outbreaks, providing important early-warning signals for public health authorities to take precautionary measures under extreme risk conditions. Separately, there were significant characteristic in the response mechanisms of FBD outbreaks to different elements, which will be analyzed in the following sections.

## Discussion

In exploring the effect of temperature on FBD outbreaks, this study noticed a significant nonlinear relationship, particularly regarding the difference between extreme high or low temperature effects. Taking the weekly average temperatures of 4 °C and 28 °C as extreme values, both of their lagged-risk evolution structures displayed a certain symmetry. Under low temperature, the lagged-risk structure presented a downhill parabola, while under high temperature, it took the form of a parabola in the opposite direction. In both antithetical conditions, the extremum point of the exposure effect was obtained near lag 2–3 weeks. Overall, the 0–4 weeks cumulative RR increased with rising temperature. Specifically, when ranging from − 5 °C to 10 °C, increases in temperature led to a relatively limited increase in the risk of FBD outbreaks; when temperature exceeded the reference value, the risk increased exponentially. This cumulative effect varied across lag periods to some extent, with the greatest cumulative effect occurring at 0–1 weeks, a significant decrease at 1–2 weeks, and a slight reversal of the pathogenic effect at 2–4 weeks. The temperature-induced pathogenicity risk showed complex multimodal dynamics over time, but its main pathogenic effect was concentrated in the lag 0–1 weeks.

This finding highlights the rapid response properties of temperature as a pathogenic factor, indicating that short-term temperature changes can significantly impact the intensity of FBD outbreaks within a week after exposure. One possible explanation is the saturation effect^28^. After that conditions were sufficient to trigger the growth, multiplication, and spread of wild mushrooms and microorganisms, there may be a spillover consequence of subsequent consecutive temperature effects. This observation was grounded in the fact that temperature changes can directly or indirectly affect the growth, spread, and host susceptibility of pathogens and vectors^33,34^. In the short term, elevated temperature can accelerate pathogen metabolism and reproduction rates. For instance, the replication cycles of bacteria and microorganisms causing diarrhea had been accelerated in warmer environments^35^, potentially leading to faster epidemic spreading. Unsuitable temperature may also reduce human resistance to disease^9^ and even reshape behavior patterns, such as increased consumption of water and reduced attention to hygiene^36^, thereby increasing the risk of infection and transmission. Additionally, temperature changes can affect food preservation^37^, facilitating pathogen accumulation and increasing the likelihood of FBD outbreaks.

Our findings are consistent with some previous studies. For example, a study on the relationship between average temperature and gastroenteritis-related hospitalizations in Spain and similarly found that the low or high temperature effects had a symmetrical structure and the same phenomenon of RR fluctuations with longer lag times^16^. A positive relationship between ambient temperature and FBD outbreaks was also consistent with the results of other studies at the week scale^26,31^. Given that this study focused on specific temperature ranges and analyzed the cumulative RR over different lag periods in detail, its findings may differ from other studies. In general, given the rapid-response nature of temperature-induced pathogenic risks, public health departments should be highly sensitive to temperature changes and implement more timely and precise public health interventions. Earlier warning mechanisms should be activated when unusual temperature events occur to reduce the temperature-induced disease burden.

In contrast to the immediate effect of heat-induced outbreaks, the pathogenic risk associated related to high precipitation moved more rearward. The results highlight that when assessing the impact of precipitation on FBD, the delayed characteristics and the complex interaction mechanisms should be fully considered. Firstly, the RR had a higher growth rate with increasing precipitation during lag 1–3 weeks. Meanwhile, extreme precipitation also maximized its threat to FBD outbreaks during this period. Subsequently, its pathogenicity decreased significantly within 1 week and after 3 weeks.

The mechanism of delayed effect may be closely associated with the characteristics of precipitation itself. Compared with temperature, although increased precipitation also affects disease transmission, for example, by creating more waterlogged areas for pathogens to colonize^38^, it may not be as responsive as temperature because temperature can directly stimulate pathogen metabolism and reproduction. Moreover, excessive precipitation may trigger secondary natural disasters such as flooding. These disasters can temporarily disrupt human activities by reducing movement, thereby decreasing the short-term risk. However, this temporary dampening effect cannot fully counteract the long-term impact of precipitation. In the long term, excessive precipitation may cause an increase in bacterial concentrations and turbidity in rivers, thus deteriorating water quality^39,40^. Meanwhile, large amounts of standing water and damaged infrastructure may provide more favorable conditions for microbial colonization and disease transmission.

When compared with existing studies, a similar finding has been reported in a case study of FBD in Singapore, where Salmonella infection cases increased 2 weeks after increased precipitation but decreased after 5 weeks^25^. Additionally, a study on Campylobacter incidence in Denmark, Finland, Norway, and Sweden also found a link with precipitation in the previous week^13^. These consistencies across different regions and pathogens reinforce the generalizability of the relationship between precipitation and FBD outbreaks. However, differences may exist in specific details due to variations in local environmental conditions, pathogen types, and study methodologies.

The results of this study revealed a negative correlation between wind speed and FBD outbreaks. The risk was significantly higher under low wind speed. This may be because low wind speeds are more conducive to the localized accumulation of microorganisms and pollutants, thereby increasing the likelihood of transmission. Moreover, the RR had some fluctuations during different lag periods, but the overall effects were similar, indicating that wind speed had an analogous influence pattern in the lag time dimension. One possible explanation is that not all foodborne pathogens can be airborne or form spores. Although viable foodborne microorganisms have been detected in air samples, the pathogenicity and transmission distance of these pathogens remain uncertain^41^.

Wind speed is a crucial factor influencing the spread of FBD, since it can modulate the dynamics of different vectors and pathogens^42^. The mechanism connecting wind speed and disease transmission is complex. One manifestation is dilution: faster wind speeds accelerate air movement, diluting the pathogen concentration, and theoretically reducing the chances of pathogen exposure^43^. Another manifestation is the accelerated transmission effect: suitable wind speeds can simultaneously facilitate the large-range transmission of pathogens, accelerating the spread rate of foodborne pathogens such as Salmonella, Staphylococcus aureus, Vibrio, and other bacterial agents across spatial scales^41^. For example, pathogenic bacteria have been detected in the air near landfills, and farms nearby may be contaminated. Additionally, wind speed modulates environmental variables like carbon dioxide and relative humidity^42^, which are important factors in analyzing disease spread.

Based on aforementioned reversed effects, existing studies have found various relationships between wind speed and diseases, such as inverted U-shape, positive or negative correlation, or even uncorrelated^41,44,45^. Studies on the effect of wind speed on FBD outbreaks are relatively under-developed. Our finding of a negative correlation was different from some of the previous results^24^. The differences may be due to differences in study areas, pathogen types, and detection methods. This indicates that when assessing the impact of wind speed, it is necessary to integrate multiple factors such as specific regions, climates, and population characteristics. Relying solely on a single factor analysis result may yield unreliable conclusions.

## Conclusion

The research utilized DLNM to combine meteorological factors with FBD outbreaks to uncover potential correlations. The results demonstrated significant nonlinear associations between meteorological conditions (such as temperature, precipitation, and wind speed) and FBD outbreaks. The lagged exposure-response patterns differed among these various meteorological elements, posing substantial challenges for public health interventions. Specifically, higher temperature, greater precipitation, and lower wind speed generally indicated a higher risk of FBD outbreaks. Considering the observed trends of climate change and extreme weather events, understanding the relationship between meteorological conditions and FBD outbreaks holds practical significance. The findings here offer insights into more comprehensively assessing the FBD outbreaks risk, and they also reiterate the importance of the early warning, prevention, and control measures implemented by public health authorities. For example, by establishing a continuous monitoring system for meteorological and environmental conditions, public health departments can release timely warning information to guide all aspects of food handling and storage, thus reducing the risk of FBD outbreaks.

The main weakness of this study was the insufficient consideration of fully confounding factors. The causative factors of FBD are complex and multifaceted, affected by multiple factors such as geographic location, population distribution, age structure, food culture, and customs^15,20^. Further experimental investigations are required to estimate more meteorological and environmental elements, such as humidity, evapotranspiration, sunshine duration, and vegetation index, which have been demonstrated to impact disease burden^28,46^. In addition, this research focuses on the overall analysis of the Yantai region. Research on districts and counties at a smaller observational scale and analyses by categories of foodborne pathogens will facilitate a better understanding of the mechanisms of FBD outbreaks.

## Data Availability

The datasets analyzed in this study are generally not publicly available due to confidentiality agreements, but are available from the corresponding author on reasonable request.
